# Biopsychosocial Predictors of Pain Persistence and Pain Chronification in Temporomandibular Disorders: A Systematic Review

**DOI:** 10.3390/jcm15072498

**Published:** 2026-03-25

**Authors:** Piotr Seweryn, Marta Waliszewska-Prosol, Marcin Derwich, Anna Paradowska-Stolarz, Magdalena Gebska, Mieszko Wieckiewicz

**Affiliations:** 1 Department of Experimental Dentistry, Wroclaw Medical University, 50-425 Wroclaw, Poland; 2 Clinical Department of Neurology, Wroclaw Medical University, 50-556 Wroclaw, Poland; 3 Department of Paediatric Dentistry, Medical University of Lodz, 90-419 Lodz, Poland; 4 Department of Maxillofacial Orthopaedics and Orthodontics, Wroclaw Medical University, 50-425 Wroclaw, Poland; anna.paradowska-stolarz@umw.edu.pl; 5 Department of Rehabilitation Musculoskeletal System, Pomeranian Medical University, 70-204 Szczecin, Poland

**Keywords:** TMD, chronic pain, pain predictors, orofacial pain, depression

## Abstract

**Background/Objectives:** Temporomandibular disorders (TMD) are common causes of orofacial pain, but their clinical course varies, with some patients developing persistent symptoms. Evidence supports a biopsychosocial model of pain chronification, yet prognostic factors for pain persistence in TMD remain insufficiently synthesized. This systematic review aimed to identify biological, psychological, and social predictors associated with pain persistence and chronicity in painful TMD. **Methods:** This review was conducted in accordance with PRISMA 2020 guidelines and registered in PROSPERO (CRD420261286566). MEDLINE, Embase, and Web of Science were searched for studies published between January 2010 and December 2025. Eligible studies included adult patients with painful TMD and assessed baseline biopsychosocial predictors of pain persistence or chronicity at follow-up ≥ 3 months. Risk of bias was assessed using QUIPS and PROBAST. Due to heterogeneity across studies, findings were synthesized narratively. **Results:** Six prospective cohort studies were included, with follow-up durations ranging from 6 to 24 months. Psychological factors, particularly pain catastrophizing and depression, were associated with increased risk of pain persistence. Higher baseline pain intensity and widespread pain also showed prognostic value. Sleep-related and behavioral factors demonstrated inconsistent associations, and social predictors were rarely examined. The certainty of evidence ranged from moderate for catastrophizing and pain intensity to very low for sleep-related and occlusal factors. **Conclusions:** Pain persistence in TMD is influenced by multiple biopsychosocial factors. Psychological variables, especially catastrophizing and depression, appear to be the most consistent predictors, although this finding should be interpreted with caution, given the small number of included studies. These findings highlight the importance of comprehensive biopsychosocial assessment in patients with painful TMD and the need for further longitudinal research.

## 1. Introduction

Temporomandibular disorders (TMD) are commonly described as a diversified group of conditions affecting the temporomandibular joints, the masticatory muscles, and surrounding tissues [1,2]. The etiology of TMD, including masticatory muscle pain as well as pain in the joints, both known as a painful TMD, is multifactorial [3,4]. According to the biopsychosocial model, there can be distinguished three major groups of factors leading to the development of TMD, namely: biological, psychological, and social factors [5].

Masticatory muscle pain is among the most frequent causes of non-odontogenic orofacial pain and is commonly encountered in daily dental and orofacial pain practice [6]. It is often regarded as a reversible and self-limiting condition, particularly at early stages. Nevertheless, longitudinal observations indicate that this assumption is not always justified. There have been reported cases in which pain persists beyond the expected period of tissue healing and gradually becomes chronic, leading to functional impairment and deterioration of quality of life [2,7,8]. Once pain persistence is established, treatment outcomes tend to be less predictable, and the overall burden for patients increases, both clinically and socially [9].

Over the last two decades, prospective cohort studies have substantially contributed to the understanding of factors associated with the onset and persistence of painful TMD [8]. In particular, findings from the Orofacial Pain: Prospective Evaluation and Risk Assessment (OPPERA) project have demonstrated that local musculoskeletal findings alone do not sufficiently explain why pain persists in some patients but not in others [2,8,9]. It has been suggested that pain chronicity develops rather as a consequence of interactions between altered pain processing, psychological vulnerability, and broader contextual or behavioral influences [2,8].

From a biological perspective, increased pain sensitivity has been repeatedly identified as a relevant factor preceding the development of painful TMD. Reduced pressure pain thresholds, widespread hyperalgesia, and other indicators of facilitated nociceptive processing have been associated with both first-onset TMD and persistence of pain over time [7,8]. These findings are commonly interpreted within the framework of central sensitization (CS) and suggest that, at least in a subgroup of patients, masticatory muscle pain reflects a more generalized disturbance of pain modulation rather than a strictly local muscle disorder. In addition, genetic and inflammatory mechanisms have been proposed to influence individual susceptibility, as associations between polymorphisms in pain- and inflammation-related genes and pain severity or persistence have been reported [10,11,12].

Psychological factors appear to contribute to the clinical course of painful TMD in a meaningful way. Prospective data indicate that pain catastrophizing, anxiety, depression, negative affect, and stress-related variables are associated with an increased risk of developing painful TMD as well as with less favorable pain trajectories over time [7,13]. These factors may influence how pain is perceived and interpreted, but also how patients respond behaviorally to pain. Results from twin and family studies further suggest that cognitive–emotional pain-related phenotypes contribute to pain chronicity beyond shared genetic background, underlining the independent role of psychological processes [14].

Social and behavioral factors have also been implicated in the chronification of painful TMD, although the available evidence is less consistent. Sleep disturbances, general health status, parafunctional behaviors, and occupational stress have been reported to be associated with painful TMD and with chronic overlapping pain conditions [15,16,17]. It has been proposed that impaired sleep may act as a nonspecific amplifier of pain sensitivity and stress responsiveness, thereby interacting with biological and psychological mechanisms involved in pain maintenance [16].

Despite growing recognition of the biopsychosocial nature of pain chronicity, the existing literature remains heterogeneous and difficult to integrate. Many studies focus on single domains or on pain onset rather than pain persistence. Moreover, cross-sectional designs are still frequently employed, limiting prognostic interpretation. Even within longitudinal studies, biological, psychological, and social predictors are often examined separately, without adequate consideration of their potential interactions.

Therefore, the aim of the present systematic review was to identify biopsychosocial predictors of pain chronicity in patients with painful TMD. By synthesizing evidence from prospective and longitudinal studies, this review seeks to clarify which baseline factors are associated with pain persistence and to contribute to a more clinically oriented, individualized understanding of chronic pain risk in this patient population.

## 2. Materials and Methods

### 2.1. Protocol and Registration

This systematic review was conducted in accordance with the Preferred Reporting Items for Systematic Reviews and Meta-Analyses 2020 (PRISMA 2020) [18]. The completed PRISMA 2020 checklist is provided in the Supplementary Materials. The protocol was registered in the PROSPERO database under the registration number CRD420261286566.

### 2.2. Eligibility Criteria

Eligibility criteria were defined according to the PICOS framework and are summarized in Table 1 [19].

The following inclusion criteria were applied:

(P) Population.

Studies involving adult participants (>18 years old) diagnosed with painful TMD were eligible, including myogenous, arthrogenic, or mixed TMD type. Studies focusing exclusively on non-painful TMD conditions (e.g., disc displacement with reduction) were excluded. A painful TMD diagnosis was preferably established using standardized diagnostic criteria, such as the Research Diagnostic Criteria for Temporomandibular Disorders (RDC/TMD), the Diagnostic Criteria for Temporomandibular Disorders (DC/TMD), or the International Classification of Orofacial Pain (ICOP). To ensure broad identification of relevant evidence, studies applying other clearly described clinical diagnostic approaches were also considered eligible, provided that painful TMD was explicitly defined.

(I/E) Prognostic factors/Exposure.

Studies investigating biological, psychological, social, or behavioral prognostic factors assessed at baseline and examined in relation to subsequent pain outcomes were eligible. Prognostic factors included, but were not limited to, psychological variables (e.g., depression, anxiety, pain catastrophizing), pain-related characteristics (e.g., baseline pain intensity, pain-related disability), sleep-related factors, behavioral factors (e.g., parafunctional activities), and demographic characteristics. Studies reporting pain persistence/chronicity outcomes without assessing prognostic factors were excluded.

(O) Outcomes.

The primary outcome was pain persistence or pain chronicity in painful TMD, requiring follow-up of at least 3 months. This threshold is consistent with the IASP/ICD-11 definition of chronic pain, which requires pain to persist for more than three months [20]. Related outcomes (e.g., transition to chronic pain, lack of remission, or persistence of clinically significant pain measured with validated instruments such as the Graded Chronic Pain Scale) were considered eligible if they reflected sustained pain over time. For the purpose of this review, the following definitions were applied: pain persistence refers to the continued presence of painful TMD symptoms at follow-up without clinically significant improvement; pain chronicity denotes the presence or progression of clinically significant pain, and transition from acute to chronic pain describes the process by which acute-onset TMD pain evolves into a chronic state, defined as pain duration exceeding three months in accordance with IASP/ICD-11 classification [20,21,22].

(S) Study design.

Eligible designs included prospective or retrospective cohort studies, case–control series, and secondary analyses of longitudinal cohorts. Purely cross-sectional studies without follow-up, case series, and case reports were excluded.

### 2.3. Search Strategy

A comprehensive literature search was conducted in the electronic databases MEDLINE, Embase, and Web of Science. These databases were selected to identify a broad range of relevant clinical and dental research on painful TMD. The search covered publications from January 2010 to December 2025 and was limited to peer-reviewed English-language articles. The grey literature was not searched because the review was intentionally limited to peer-reviewed studies with established methodological rigor and transparent reporting, to minimize the risk of bias arising from insufficient quality control and to enhance the comparability and reliability of the clinical conclusions. The databases were last searched on 5 January 2026. The selected time frame (2010–2025) was chosen to capture a period of substantial advances in pain research, particularly in chronic pain, as well as increasing standardization of TMD diagnostic frameworks and outcome measures. This improved methodological comparability across studies and ensured that the included evidence aligns with current concepts and assessment approaches.

Search terms combined controlled vocabulary (MeSH/Emtree) and free-text keywords; no study design filters were applied. The full database-specific search strategies are provided in Table 2.

### 2.4. Selection Process

All records identified through the database searches were imported into Rayyan QCRI (Qatar Computing Research Institute, Doha, Qatar, and Rayyan Systems, Cambridge, MA, USA) for duplicate removal and study screening [23]. Two reviewers (P.S. and M.D.) independently screened titles and abstracts to assess eligibility. Potentially relevant studies were retrieved in full text and evaluated by the same reviewers.

Throughout the screening process, reviewers were blinded to each other’s decisions. Any disagreement at either the title/abstract or full-text screening stage was resolved by consensus through discussion.

### 2.5. Data Collection

Data extraction was performed independently by two reviewers (P.S. and M.D.) using a predefined data extraction form. Reviewers were blinded to each other’s extracted data. Any discrepancies were resolved through consensus after discussion.

For each included study, the following data were extracted: study characteristics (authors, year of publication, study design), sample characteristics (sample size at baseline and follow-up, age, sex), diagnostic criteria for painful TMD (e.g., RDC/TMD, DC/TMD, ICOP or other clearly described criteria), TMD type (myogenous, arthrogenic, mixed), and pain characteristics (definition of chronic or persistent pain, baseline pain duration).

In addition, information on outcomes (outcome definition and outcome measures, e.g., pain intensity or Graded Chronic Pain Scale), baseline prognostic factors analyzed, and the statistical model used was extracted.

### 2.6. Risk of Bias Assessment

Risk of bias was assessed using the Quality In Prognosis Studies (QUIPS) tool [24]. Each included prognostic factor study was evaluated across six domains: study participation, study attrition, prognostic factor measurement, outcome measurement, study confounding, statistical analysis, and reporting.

For studies that involved the development or validation of multivariable prediction models, risk of bias was assessed using the Prediction model Risk Of Bias ASsessment Tool (PROBAST), in accordance with current methodological recommendations for prediction model studies [25].

Risk of bias assessments were performed independently by two reviewers (P.S and M.D), who were blinded to each other’s judgments. Any disagreements were resolved by consensus through discussion.

For QUIPS-based assessments, an overall risk-of-bias judgment was derived from domain-level ratings. Studies were rated as having a high overall risk of bias when at least one critical domain (e.g., study confounding or outcome measures) was judged to be high risk. When high risk of bias was identified only in non-critical domains, the overall risk of bias was considered moderate to high.

PROBAST assessments were reported according to the four domains of the tool (participants, predictors, outcome, and analysis), and the overall risk of bias judgment followed PROBAST guidance [25].

### 2.7. Data Synthesis

Due to substantial clinical and methodological heterogeneity across studies regarding study populations, definitions of pain persistence or chronicity, prognostic factors assessed, follow-up durations, and statistical approaches, a quantitative meta-analysis was not performed.

Findings were synthesized using a narrative synthesis, supported by structured tables summarizing study characteristics, outcomes, and prognostic factors.

## 3. Results

### 3.1. Study Selection

**Figure 1:**
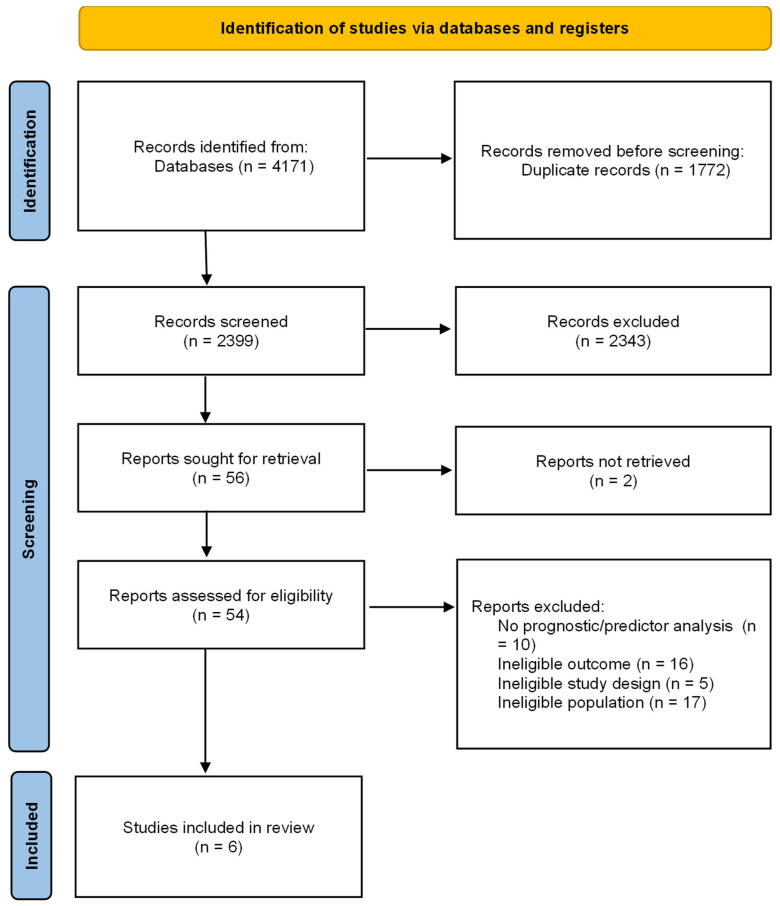
The PRISMA 2020 flow diagram.

The database search yielded 4171 records. After removal of duplicates, titles and abstracts were screened, followed by full-text assessment of potentially eligible articles. Six studies met the predefined inclusion criteria and were included in the systematic review [22,26,27,28,29,30]. The study selection process is presented in the PRISMA 2020 flow diagram (Figure 1).

### 3.2. Study Characteristics

The characteristics of the included studies are summarized in Table 3 and Table 4. All included studies had a prospective observational design and assessed pain persistence or pain chronicity in patients with painful TMD. Across studies, baseline sample size varied from 63 to 480 participants. Follow-up time ranged between 6 and 24 months. All included studies used the Research Diagnostic Criteria for Temporomandibular Disorders. Outcomes focused on pain persistence or progression to clinically significant pain, and were measured using validated tools such as the Graded Chronic Pain Scale (GCPS), Characteristic Pain Intensity (CPI), or on the basis of sustained painful symptoms. Advanced statistical models were employed (e.g., regression-based analyses) for predictor analyses.

Across the included studies, the identified predictors could be broadly grouped into three domains consistent with the biopsychosocial framework. Biological predictors included pain-related characteristics (e.g., baseline pain intensity, widespread pain). Psychological predictors encompassed cognitive and affective variables (e.g., depression, pain catastrophizing, anxiety). Social and behavioral predictors related to illness behavior and daily functioning (e.g., healthcare-seeking behavior, functional disability, sleep-related factors). A full overview of predictors analyzed across studies is presented in Table 4.

### 3.3. Risk of Bias

A summary of the risk of bias assessment is presented in Table 5.

In the included prognostic factor studies, the risk of bias across most domains was judged to be low to moderate. The domains of prognostic factor measurement and outcome measurement were consistently rated as low risk of bias, due to the use of standardized diagnostic criteria (RDC/TMD) and other validated measurement tools. Higher risk of bias was more frequent in the domains of study attrition and study confounding, primarily due to loss to follow-up and confounding in multivariable analyses.

In PROBAST analysis, the one study assessed demonstrated concerns mainly in the domain of outcome and analysis, including a lack of external validation and potential risk of overfitting.

The overall methodological quality of the included studies was considered sufficient to support qualitative synthesis of prognostic findings.

### 3.4. Certainty of Evidence

The certainty of evidence for associations between biopsychological predictors and pain persistence or chronicity was assessed using the Grading of Recommendations Assessment, Development, and Evaluation (GRADE) approach. Although GRADE was originally developed for intervention research, its application to prognostic studies is supported by published methodological guidance and has been increasingly adopted in systematic reviews of prognostic factors [31,32]. Overall, the certainty of the evidence was judged to be moderate to very low, depending on the predictor. Higher certainty ratings were observed for selected psychological and pain-related factors, whereas evidence for sleep-related and occlusal factors was rated as low or very low. Downgrading was most commonly caused by risk of bias, inconsistency across studies, and imprecision. Only predictors supported by sufficiently comparable data were included in the GRADE assessment. A summary of the findings is presented in Table 6.

### 3.5. Definitions of Pain Persistence and Pain Chronicity 

Across the included studies, outcomes related to pain persistence and chronicity were defined using similar but not identical definitions. Pain persistence was often defined as the continued presence of painful TMD symptoms at follow-up or as the lack of improvement over time [26,27,28]. Pain chronicity refers to the presence or progression of clinically significant pain [26,30]. Pain significance was often validated using tools such as the Graded Chronic Pain Scale (GCPS), or part of it, namely Characteristic Pain Intensity (CPI) [28,29,30]. In some studies, persistence or chronicity was based on sustained pain during repeated follow-up assessments [27,29]. Follow-up varied from 6 to 24 months.

The definitions of pain chronicity and pain persistence overlapped substantially, as both referred to the continued presence of painful TMD symptoms over time and were analyzed using similar tools.

## 4. Discussion

### 4.1. Summary of Main Findings

This systematic review synthesized evidence from prospective observational studies on predictors of pain persistence and chronicity in TMD. Across the six included studies, prognostic factors were heterogeneous and contained biological, psychological, social, and behavioral aspects. Overall, psychological factors, i.e., depression and catastrophizing, were associated with pain persistence or pain chronicity. Biological factors, including baseline pain intensity, number of pain conditions, and baseline number of disability days, also demonstrated prognostic value.

Social and behavioral factors were assessed less frequently and were mainly captured through indicators of illness behavior and functional impairment rather than through direct social variables. In this domain, functional disability influencing the ability to work and health care seeking were the most relevant factors associated with pain persistence or progression.

The certainty of evidence ranged from moderate to very low, reflecting methodological heterogeneity, limited sample sizes, and variability in outcome definitions across studies. In particular, the variability in outcome definitions across studies may have introduced inconsistencies in the reported findings. Some studies defined pain persistence as the continued presence of symptoms at follow-up, while others used validated tools such as GCPS to analyze the clinically significant pain. These differences limit direct comparison of results across studies and should be considered when interpreting the overall pattern of identified predictors.

To make interpretation easier, predictors identified in the included studies were grouped according to the biopsychosocial framework into three domains: biological, psychological, and social or behavioral factors. This structure allowed a clearer comparison of predictors across studies and helped identify which domains showed the most consistent prognostic associations with persistent TMD pain.

In summary, these findings support the biopsychosocial model of pain persistence and pain chronicity in patients with TMD while highlighting the need for prospective studies that more precisely analyze social and behavioral mechanisms in prognosis models.

### 4.2. Biological Predictors of Pain Persistence and Chronicity

Across the included studies, biological predictors of pain persistence and pain chronicity in painful TMD were mostly related to baseline pain characteristics, pain intensity, and pain distribution on other sites of the body. Physiological measures of pain were not employed. In the study by Velly et al., higher baseline pain intensity was associated with the onset and progression of clinically significant pain at 18-month follow-up [30]. It was still valid after adjustment for psychological factors and measures of widespread pain. Similarly, Marklund et al. reported that higher baseline pain was associated with persistence of TMD pain in the two-year period [27]. Therefore, it emphasizes the importance of the initial pain factor in the chronification process.

The distribution of pain outside of the orofacial region was also an important biological factor in pain persistence. Both Velly et al. and Su et al. identified the presence of widespread pain or pain elsewhere in the body as a predictor of pain persistence or progression [29,30]. A similar pattern has been observed in previous cross-sectional studies in chronic TMD patients, in which higher pain intensity and greater pain-related disability were associated with higher overall symptom burden [33,34,35]. These findings may demonstrate that chronic pain in TMD patients is related to alterations in pain processing and not only a localized musculoskeletal disorder. Such conclusions are in line with the concept of enhanced central pain amplification or reduced pain inhibition, although tools for CS were not employed in the included studies [33,36,37].

In a broader context outside of TMD, studies in other pain conditions suggest that baseline pain intensity and pain in other body sites are among the most consistent clinical predictors of pain persistence and long-term disability, regardless of the primary diagnosis [35,38]. Patients with chronic musculoskeletal and widespread pain with higher initial pain severity and greater area of body affected by pain, present poorer prognosis [38]. In this context, identified biological predictors in painful TMD patients align with the literature regarding chronic pain in general, where pain intensity and multisite pain act as markers of altered pain processing, rather than specific condition mechanisms [35,36]. This similarity may indicate that painful TMD shares common predictors with other chronic pain conditions.

Interestingly, functional parameters of the temporomandibular region showed some interesting results. A study by Su et al. reported that lower mandibular function impairment at baseline was associated with a higher likelihood of pain persistence at follow-up [29]. This finding may indicate that some patients experience persistent pain despite relatively preserved jaw function, suggesting that pain persistence in these cases is driven more by central mechanisms than by local functional impairment.

What is worth noting is that objective neurophysiological measures, such as quantitative sensory testing, were rarely incorporated, and their prognostic value for pain persistence was not clearly established, which limits the strength of the evidence regarding biological predictors in painful TMD. Biological factors were primarily assessed using self-reported pain tools such as GCPS [22,26,29,30]. Although measures such as pressure pain thresholds and other quantitative sensory testing parameters were assessed in one included study, they were not consistently identified as prognostic predictors, limiting the ability to analyze the relationship between biological predictors and pain-processing mechanisms, such as CS.

Therefore, although the biological predictors identified in TMD are consistent with those observed in other chronic pain conditions, further research is needed to better understand the underlying pathophysiological process, not only the patient’s self-reported pain experience.

### 4.3. Psychological Predictors of Pain Persistence and Pain Chronicity

In the included studies, psychological factors were examined more frequently and more precisely than biological aspects. Analyzed parameters encompassed psychological measures such as depression, anxiety, catastrophizing, somatization, quality of life, and perceived stress [26,28,29,30]. Psychological variables were typically assessed using validated, standardized self-report questionnaires.

Results of the studies demonstrated that pain catastrophizing (defined as a maladaptive cognitive-affective response to pain characterized by rumination, magnification and helplessness) plays an important role in unfavorable pain trajectories [30,39]. In the study by Velly et al., higher baseline levels of catastrophizing were associated with both the onset and progression of clinically significant pain at follow-up, even after adjustment for demographic variables and baseline pain intensity [30]. A similar relationship was also found for catastrophizing and pain-related disability at follow-up [30]. These findings may suggest that catastrophizing acts as an amplifier that affects pain perception and coping and can contribute to pain maintenance in TMD patients. Consistent with these findings, Forssell et al. reported that higher perceived ability to control pain can have a protective effect and was associated with more favorable outcomes [26]. It supports the thesis that the ability to cope with pain can predict long-term pain-related outcomes [26].

Interestingly, depression was also an important predictor of pain progression and disability, but after adjustment for pain catastrophizing and baseline pain-intensity it did not remain significant, while catastrophizing remained a predictor for pain outcomes [30].

Other psychological aspects showed less consistent results with pain persistence. Anxiety and psychological distress were examined in three studies, but their predictive value was not significant after adjustment for pain-related variables and comorbid conditions [26,27,29]. Findings from the OPPERA cohort confirm the dynamic relationship between psychological factors and pain persistence. Specifically, a study by Ohrbach et al. demonstrated that psychological distress may vary, and it tends to increase around TMD onset and is elevated in patients with persistent pain, but is declining in those whose symptoms are decreasing [28]. These findings support a dynamic model of the bidirectional relationship between psychological distress and TMD pain.

Chronic pain was previously associated with psychological disorders, particularly with depression and anxiety symptoms, which were also recognized as predictors of chronic pain conditions [40]. In a study by Aaron et al., approximately 40% of adults with chronic pain had significant depression and anxiety symptoms [41]. Interestingly, greater severity of psychological disorders was observed in participants with nociplastic pain conditions, such as fibromyalgia, compared with patients presenting nociceptive pain mechanisms (i.e different types of arthritis) [41]. It supports the view that centrally mediated pain is closely related to psychological impairment.

These observations are important in the context of painful TMD, where chronic myogenous pain was linked to altered pain processing mechanisms. Studies in patients with chronic masticatory muscle pain demonstrated a strong association between depression and anxiety symptoms and markers of CS, suggesting that psychological factors may be related to central nervous system alterations and not only the tissue damage [33].

Chronic pain and psychological disorders, such as depression, involve overlapping brain regions such as the hippocampus, thalamus, and amygdala, which are responsible for emotional regulation but also for pain processing [42,43].

The findings regarding psychological factors indicate that baseline psychological aspects, especially catastrophizing and depression, may be a relevant clinical predictor of pain persistence in painful TMD. It highlights the importance of assessing psychological factors during patient evaluation and supports the biopsychosocial model of understanding pain chronification.

### 4.4. Social and Behavioral Predictors of Pain Persistence and Pain Chronicity

In the biopsychosocial framework, the social domain refers to how patients function in their social context, including daily roles, work, interpersonal functioning, and healthcare use. Classical social determinants such as socioeconomic status, education, or social support were not directly assessed in the prospective studies included in the review. Therefore, “social predictors” in this analysis mainly refer to proxies of social functioning and illness behavior rather than direct measures of social context.

Four domains were identified: previous healthcare visits, healthcare utilization, functional disability impacting work or social life, and socio-behavioral stressors. Overall, social predictors were rarely investigated heterogeneously, and only two domains showed consistent prognostic relevance.

Regarding previous healthcare visits, only one study demonstrated that earlier healthcare contact may predict later pain persistence. Forssell et al. evaluated the number of pain-related healthcare visits in the six months preceding baseline. Each additional visit significantly increased the risk of clinically significant pain after one year, and this variable remained significant in multivariable analysis [26]. This finding was interpreted as reflecting frequent healthcare-seeking behavior, possibly indicating more complex symptom presentation or greater distress.

In contrast, Su et al. recorded previous treatment for TMD pain as a binary variable, which did not predict persistence at 6 or 12 months [29]. In OPPERA, participants with prior diagnosis or treatment were excluded, and in Marklund, Velly, and Elsaraj, healthcare history was not analyzed as a prognostic factor [22,27,28,30]. These discrepancies may partly reflect methodological differences, particularly the use of continuous versus dichotomous indicators.

Regarding general healthcare utilization, none of the included prospective studies formally evaluated it as an independent predictor of chronic pain. Although utilization was mentioned in several studies, no cohort included detailed quantitative measures, such as the number of consultations or treatment intensity, in prognostic models [26,28,29]. Consequently, current evidence does not allow conclusions regarding whether healthcare utilization per se predicts persistence of painful TMD.

Functional disability interfering with work and social activities emerged as the most consistent social predictor. Velly et al. used the disability component of the Graded Chronic Pain Scale, which includes interference with daily occupational and social activities [30]. Baseline pain-related disability significantly predicted both the onset and the progression of clinically significant chronic pain over 18 months. Higher initial interference increased the risk of developing more severe pain grades.

In other studies, functional limitation was assessed mainly in clinical terms. Ohrbach analyzed jaw functional limitation and chewing difficulties, and Su et al. used the Mandibular Function Impairment Questionnaire; however, these measures reflected stomatognathic function rather than work or social participation [28,29]. Consequently, only Velly et al. directly evaluated the prognostic role of work- and social-related disability [30]. Finally, socio-behavioral stressors were not systematically assessed as independent prognostic predictors of pain chronicity. Most cohorts focused primarily on psychological constructs such as depression, anxiety, or catastrophizing. Although some authors discussed potential social mechanisms, such variables were not included in statistical models. Therefore, current evidence does not support an independent prognostic role of broader social stressors in painful TMD.

In summary, prospective studies indicate that the social component of the biopsychosocial model has been only partially investigated in painful TMD. Among the selected domains, only two constructs showed prognostic relevance: frequent early healthcare-seeking behavior and functional disability interfering with work and social activities. Importantly, these variables serve as proxies for illness behavior and social functioning rather than classical social determinants. In contrast, general health care utilization and socio-behavioral stressors were either not assessed or not consistently associated with the outcome.

These findings indicate that social predictors of chronicity are mainly related to early maladaptive illness behavior and to the social consequences of pain rather than to external stress exposures. At the same time, the scarcity of positive results highlights an important gap in current research. Most studies concentrate on biological and psychological mechanisms, while the social dimension of chronic pain remains underrepresented. Future longitudinal studies should systematically include measures of occupational functioning, social participation, healthcare-seeking patterns, and social support in order to better understand how social context contributes to the persistence of painful TMD.

### 4.5. Clinical Implications

The findings of this systematic review have important implications for clinical practice regarding the assessment and treatment of patients with painful TMD. The identified psychological factors, such as pain catastrophizing and depression, highlight the importance of including psychological aspects in the daily routine in the management of painful TMD. Standardized screening tools, such as validated questionnaires, may help identify patients at higher risk of unfavorable pain trajectories.

Higher baseline pain intensity and widespread pain also demonstrated prognostic value, pointing out that assessment should consider regions outside of the orofacial area. It can also suggest possible alterations in pain processing.

Behavioral factors, such as parafunctional activities or sleep disturbances, may also contribute to pain persistence, although current evidence is limited and less consistent.

Early identification of prognostic factors may help to plan more appropriate and effective treatment. It should include patient education, behavioral changes, and interdisciplinary management.

### 4.6. Limitations of the Review and Future Research Directions

This review has several limitations. First, the number of included studies was relatively small, which limits the strength and generalizability of the conclusions. Second, high heterogeneity was observed in outcome definitions, follow-up duration, and the predictors assessed, making it impossible to perform a meta-analysis. In particular, follow-up periods ranged from 6 to 24 months, which may have influenced the findings; shorter follow-up periods may not capture late onset chronification, whereas longer follow-up introduces greater risk of attrition and changes in clinical management over time. Third, the certainty of evidence ranged from moderate to very low for most predictors. In addition, most studies relied primarily on self-reported measures, while objective physiological or neurophysiological indicators were rarely assessed and showed inconsistent prognostic value. Fourth, grey literature was not searched, which may introduce publication bias and limit the scope of the identified evidence. Fifth, the single study evaluated using PROBAST showed concerns regarding external validation and risk of overfitting, which further limits the generalizability of its findings.

Future studies should use standardized outcome definitions, objective pain measures, and comprehensive biopsychosocial assessments to improve the prognostic accuracy.

## 5. Conclusions

This systematic review showed that pain persistence in painful TMD is influenced by multiple biopsychosocial factors. Psychological variables, especially pain catastrophizing and depression, represent predictors of unfavorable pain outcomes, although this conclusion is limited by the small number of included studies and heterogeneity across outcome definitions. Biological factors, such as higher baseline pain intensity and widespread pain, also contribute to increased risk of persistent pain.

These findings support the biopsychosocial model of pain chronification and highlight the importance of comprehensive assessment beyond local musculoskeletal factors.

Further longitudinal studies using standardized outcome definitions and comprehensive biopsychosocial assessment are needed to improve prognostic models and support targeted preventive strategies.

## Figures and Tables

**Table 1:** Inclusion and exclusion criteria.

	Inclusion Criteria	Exclusion Criteria
Population	Adults (≥18), painful TMD	<18, non-painful TMD only, painful TMD not defined
Prognostic factors/Exposure	Baseline biopsychosocial/behavioral factors	No predictor/prognostic factor analysis
Comparator	Persistent/chronic pain vs. improved/remitted pain (within-study)	No within-study comparison for pain persistence/chronicity
Outcomes	Pain persistence/chronicity; follow-up > 3 months	Follow-up < 3 months, outcomes unrelated to pain persistence,
Study design	Cohort (prospective/retrospective), case–control	Cross-sectional, case series, case reports

**Table 2:** Search strategies for each database.

Database	Search String
Medline	(“Temporomandibular Disorders”[Mesh] OR “temporomandibular disorder*”[tiab] OR TMD[tiab] OR “temporomandibular joint” OR “temporomandibular joint disorder” OR “myogenous TMD”[tiab] OR “myogenic TMD”[tiab] OR “masticatory muscle pain”[tiab] OR “masticatory myalgia”[tiab] OR “muscle related TMD”[tiab] OR “myofascial pain”[tiab] OR “jaw muscle pain”[tiab]) AND (chronic*[tiab] OR persistent[tiab] OR “persistent pain”[tiab] OR “pain persistence”[tiab] OR “pain duration”[tiab] OR “follow-up”[tiab] OR longitudinal[tiab] OR prospective[tiab] OR cohort[tiab]) AND (predictor*[tiab] OR “risk factor*”[tiab] OR prognos*[tiab] OR determinant*[tiab] OR associated[tiab] OR baseline[tiab])
Embase	(‘temporomandibular disorder’/exp OR ‘temporomandibular disorder*’:ti,ab OR TMD:ti,ab OR ‘temporomandibular joint’:ti,ab OR ‘temporomandibular joint disorder*’:ti,ab OR ‘myogenous TMD’:ti,ab OR ‘myogenic TMD’:ti,ab OR ‘masticatory muscle pain’:ti,ab OR ‘masticatory myalgia’:ti,ab OR’ myofascial pain’:ti,ab OR ‘jaw muscle pain’:ti,ab) AND (chronic*:ti,ab OR persistent:ti,ab OR ‘persistent pain’:ti,ab OR ‘pain persistence’:ti,ab OR ‘pain duration’:ti,ab OR ‘follow-up’:ti,ab OR longitudinal:ti,ab OR prospective:ti,ab OR cohort:ti,ab) AND (predictor*:ti,ab OR ‘risk factor*’:ti,ab OR prognos*:ti,ab OR determinant*:ti,ab OR associated:ti,ab OR baseline:ti,ab)
Web of Science	TS = ( (“temporomandibular disorder*” OR TMD OR” temporomandibular joint” OR “myogenous TMD” OR “myogenic TMD” OR “masticatory muscle pain” OR “masticatory myalgia” OR “myofascial pain” OR “jaw muscle pain”) AND (chronic* OR persistent OR “persistent pain” OR “pain persistence” OR “pain duration” OR “follow-up” OR longitudinal OR prospective OR cohort) AND (predictor* OR “risk factor*” OR prognos* OR determinant* OR associated OR baseline))

**Table 3:** Studies characteristics.

l.p	Author	Year	Study Design	Sample Size (Baseline)	Sample Size (Follow-Up)	Age (Mean ± SD/Range)	Diagnostic Criteria	TMD Diagnosis Subtype	Pain Duration at Baseline	Outcome Type (Pain Persistence, Pain Chronicity)	Chronic Pain/Pain Persistence Definition	Outcome Measure (e.g., Pain Intensity, GCPS)
1	Elsaraj et al.	2023 [22]	prospective cohort study	456	378	41.74 ± 16.29	RDC/TMD and DC/TMD	painful TMD (muscle and/or joint)	acute cohort < 3 months; chronic > 3 months	Pain chronification (transition from acute to chronic) and persistence of chronic painful TMD	Pain duration > 3 months; Dysfunction GCPS II-IV	Pain persistence at 3-month follow-up (pain duration > 3 months), GCPS (1 vs. II-IV), Characteristic pain intensity
2	Ohrbach et al.	2020 [28]	prospective cohort study	260	147	N.D	RDC/TMD	Painful TMD	No TMD at baseline	Pain persistence	Persistence defined as examiner-verified TMD at 6–8 months after onset	Examiner-verified presence of symptoms of TMD
3	Forssell et al.	2016 [26]	prospective cohort study	399	263	40.5 ± 12.7	RDC/TMD	Painful TMD	TMD pain in last month	Pain persistence	pain persistence at 1 year follow-up (according to GCPS)	GCPS at 1 year
4	Velly et al.	2011 [30]	prospective cohort study	570	480	35.85 ± 12.48	RDC/TMD	Painful TMD	>3 months	Pain persistence/chronicity	pain frequency of at least once per week, and duration of at least 3 months	GCPS-CPI
5	Marklund et al.	2010 [27]	case–control with 2-year prospective cohort	371	280	N.D	RDC/TMD	Painful TMD	N.D	Pain persistence	2 years of pain persistence	Persistence of painful symptoms at 0, 12 and 24 months
6	Su et al.	2020 [29]	prospective cohort study	63	63	40.5 ± 13.9	RDC/TMD	Myofascial pain	pain for at least 1 month	pain persistence	Pain persistence in the 6 and 12-month follow-up	GCPS-CPI

**Table 4:** Predictors of pain persistence and chronicity in temporomandibular disorders.

l.p	Author	Predictors Analyzed (e.g., Somatization, Number of Painful Sites)	Statistical Model (Logistic Regression, Linear Regression)	Significant Predictors
1	Elsaraj et al. [22]	EDS, Insomnia, PHQ-4, Characteristic Pain Intensity, Baseline acute/chronic status, GCPS	Logistic regression	No associations between insomnia and persistence or transition to chronic pain, Borderline association between transition or persistence risk and EDS,
2	Ohrbach et al. [28]	Psychosocial measures (perceived stress/negative life events, anxiety, mood/affect, depression, somatic symptom reporting, pain catastrophizing), Sleep quality and health-related quality of life, Oral parafunctional behaviors and jaw functional limitation, Quantitative sensory testing, pressure pain thresholds and thermal/mechanical pain response	Logistic and linear change	None reported
3	Forssell et al. [26]	Comprehensive multidimensional pain questionnaire assessing TMD pain related and general health factors, and psychosocial prognostic factors using validated self-report scales.	Logistic regression	Number of healthcare visits (last 6 months), Pain intensity/dysfunction of other pain conditions, Number of other pain conditions: (borderline), Number of disability days (baseline), Perceived ability to control pain (borderline)
4	Velly et al. [30]	Catastrophizing, Depression, Widespread pain, Worst pain intensity, GCPS at baseline,	Linear regression, binary logistic regression, ordinal logistic regression	Baseline catastrophizing, Pain intensity at baseline, Widespread pain,
5	Marklund et al. [27]	Self-reported bruxism, Dental occlusal factors, Parafunctions,	Logistic regression	Self-reported bruxism, Crossbite, Mandibular instability in the Intercuspal Position, Lateral slide 1 mm between the retruded contact position and the Intercuspal Position
6	Su et al. [29]	Pain characteristics (baseline Characteristic Pain Intensity, pain duration/chronicity, pain elsewhere), Prior TMD pain treatment, Psychosocial factors (SCL-90 depression and somatization), Bruxism, Mandibular function impairment	Logistic regression	Pain elsewhere Depression, Bruxism, Mandibular Function

(EDS—Excessive Daytime Sleepiness, PHQ-4—Patient Health Questionnaire 4, GCPS—Graded Chronic Pain Scale, TMD—Temporomandibular Disorders, SCL-90—Symptoms Checklist 90).

**Table 5:** Risk of bias analyzed with QUIPS tool.

Author	Study Participation	Study Attrition	Prognostic Factor Measurement	Outcome Measurement	Study Confounding	Statistical Analysis and Reporting	Overall Bias
Elsaraj et al. [22]	Moderate	Moderate	Low	Low	High	Moderate	High
Ohrbach et al. [28]	Low	High	Low	Low	Moderate	Moderate	Moderate
Forssell et al. [26]	Moderate	Moderate	Moderate	Moderate	Moderate	Moderate	Moderate
Velly et al. [30]	Low	Moderate	Low	Low	Moderate	Low	Moderate
Markulund et al. [27]	High	Moderate	Low	Low	High	High	High

**Table 6:** GRADE assessment.

Prognostic Factor	No. of Studies (First Author)	Total N	Outcome Definition	Effect Estimate (Adjusted)	Certainty of Evidence (GRADE)
Catastrophizing	2 (Velly; Forssell)	~740	GCPS II–IV/progression or persistence	OR 1.72–2.16	⬤⬤⬤◯ Moderate
Baseline pain intensity	2 (Velly; Forssell)	~740	GCPS II–IV/disability	Dose–response relationship	⬤⬤⬤◯ Moderate
Depression	2 (Velly; Forssell)	~700	GCPS II–IV/persistence	OR ~1.2–1.5	⬤⬤◯◯ Low
Widespread pain/pain elsewhere	2 (Velly; Forssell)	~700	GCPS II–IV/persistence	OR ~1.3–1.8	⬤⬤◯◯ Low
Excessive daytime sleepiness (EDS)	1 (Elsaraj)	378	Pain > 3 months/GCPS II–IV	RR 1.13 (1.00–1.26)	⬤◯◯◯ Very low
Bruxism/occlusal factors	1 (Marklund)	280	2-year persistence	Inconsistent effects	⬤◯◯◯ Very low

⬤⬤⬤⬤ High, ⬤⬤⬤◯ Moderate, ⬤⬤◯◯ Low, ⬤◯◯◯ Very low.

## Data Availability

No new data were created or analyzed in this study.

## Supplementary Materials

The following supporting information can be downloaded at: https://www.mdpi.com/article/10.3390/jcm15072498/s1, PRISMA 2020 Checklist.

## Abbreviations

The following abbreviations are used in this manuscript:

TMD	Temporomandibular Disorders
TMJ	Temporomandibular Joint
DC/TMD	Diagnostic Criteria for Temporomandibular Disorders
RDC/TMD	Research Diagnostic Criteria for Temporomandibular Disorders
ICOP	International Classification of Orofacial Pain
PRISMA	Preferred Reporting Items for Systematic Reviews and Meta-Analyses
PICOS	Population, Intervention/Exposure, Comparator, Outcome, Study Design
PROSPERO	International Prospective Register of Systematic Reviews
QUIPS	Quality In Prognosis Studies
PROBAST	Prediction model Risk Of Bias ASsessment Tool
GRADE	Grading of Recommendations Assessment, Development, and Evaluation
GCPS	Graded Chronic Pain Scale
CPI	Characteristic Pain Intensity
EDS	Excessive Daytime Sleepiness
ISI	Insomnia Severity Index
PHQ-4	Patient Health Questionnaire-4
SCL-90	Symptom Checklist-90
CS	Central Sensitization
OR	Odds Ratio
RR	Risk Ratio
CI	Confidence Interval
